# SSA‐ZP on Scalp Seborrheic Dermatitis: Regulating Sebum Levels and Scalp Barrier

**DOI:** 10.1111/jocd.16617

**Published:** 2024-10-28

**Authors:** Qi Wang, Yufang Liu, Congxiu Ye, Jing Shen, Jiahui Lin, Yunfan Du, Lintong Li, Xiaowen Huang, Yue Zheng

**Affiliations:** ^1^ Department of Dermatology Nanfang Hospital, Southern Medical University Guangzhou China; ^2^ Department of Dermatology and Venereology Fuyang People's Hospital Fuyang China; ^3^ Department of Dermatology The Third Affiliated Hospital of Sun Yat‐Sen University Guangzhou China

**Keywords:** scalp barrier function, scalp seborrheic dermatitis, sebum level, supramolecular salicylic acid, zinc pyrithione

## Abstract

**Background:**

The occurrence of scalp seborrheic dermatitis (SD) is closely related to the production of sebum and scalp barrier function. Supramolecular salicylic acid has the keratolytic and anti‐inflammatory properties, and zinc pyrithione is an antifungal drug delivered as a microparticle to skin epithelia.

**Objective:**

This study aimed to assess the efficacy and safety of 2% supramolecular salicylic acid (SSA) combined with 0.8% zinc pyrithione conditioner (SSA‐ZP) in treating scalp SD focusing on key outcome measures including sebum levels and scalp barrier function.

**Methods:**

Five patients with mild to moderate scalp SD were included in the 2‐week self‐controlled prospective study, receiving daily SSA‐ZP treatment. Evaluation on days 0, 7, and 14 included dermoscopy, the adherent scalp flaking scale (ASFS), sebum level, transepidermal water loss (TEWL), pH, and stratum corneum hydration. We also performed the fungal count across specific scalp regions, such as the left and right sides of the forehead, the top of the head, and the occiput.

**Results:**

Five patients with mild to moderate scalp SD participated in this study. After 2 weeks of SSA‐ZP treatment, significant reductions in ASFS scores, sebum levels, and fungal count were observed, alongside improvements in TEWL and pH values across multiple scalp regions. Moreover, there was no difference in the hydration of stratum corneum.

**Conclusion:**

SSA‐ZP demonstrated efficacy in treating scalp SD without adverse effects, suggesting its potential as a first‐line treatment option. Further research with larger sample sizes and longer follow‐up periods is warranted to validate these findings.

## 1 Introduction

Scalp seborrheic dermatitis (SD) is a chronic skin condition influenced by fungal infection, sebum production, and inflammatory factors [1, 2], leading to its recurrent nature. The intricate interplay between Malassezia, sebum production, and the immune responses contributes to the onset and recurrence of scalp SD [3, 4].

Salicylic acid has been widely used in treating dermatoses, such as acne and seborrheic dermatitis. Supramolecular salicylic acid (SSA) from a medical perspective primarily focuses on utilizing the self‐assembly properties of salicylic acid molecules to enhance drug delivery systems, improve stability, and target specific sites within the body. SSA, previously employed in treating facial acne, has demonstrated efficacy in mitigating inflammatory erythema and reducing sebum secretion without compromising the skin barrier [5].

Zinc pyrithione (ZP) serves as a traditional antifungal agent, effectively addressing scalp dandruff and itchiness [6, 7]; however, its lack of impact on inflammation and sebum secretion often leads to frequent relapse of scalp SD [8].

Two percent supramolecular salicylic acid (SSA) combined with 0.8% zinc pyrithione conditioner (SSA‐ZP) is a topical medication which was approved in China [9]. The combined application of SSA and ZP for scalp SD remains unexplored. Thus, our study endeavors to conduct a prospective self‐controlled clinical trial, assessing the efficacy and safety of SSA in conjunction with ZP conditioner for treating scalp SD.

## 2 Materials and Methods

### 2.1 Study Design

**FIGURE 1:**
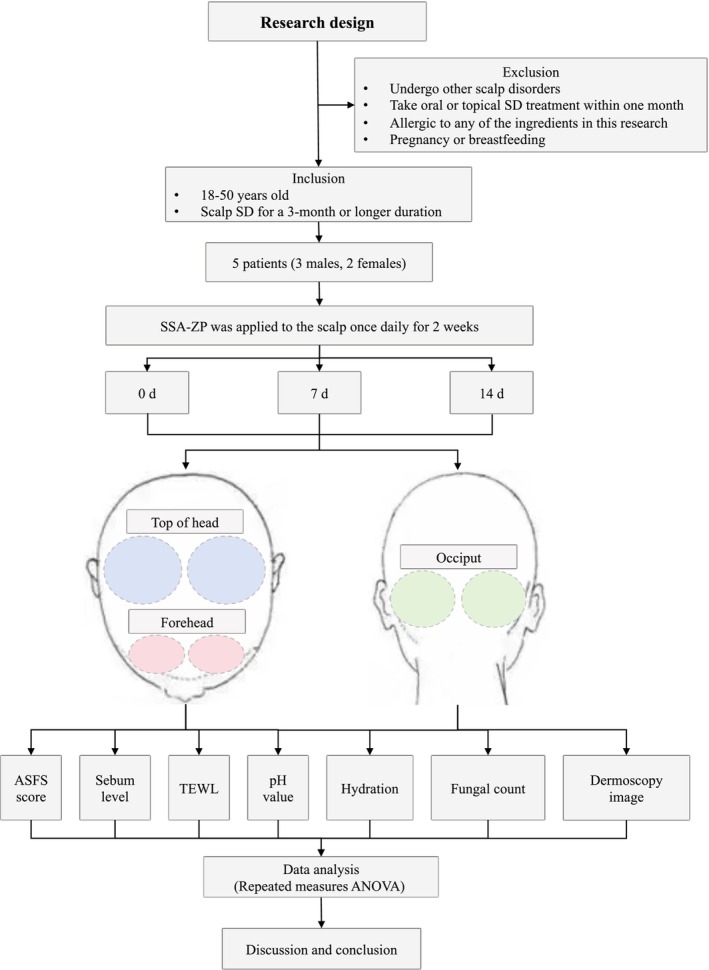
Flow schematic of the research design. Flow schematic demonstrating the study design, encompassing patient selection, treatment procedures, skin parameter measurements, and data analysis. ANOVA, analysis of variance; ASFS, the adherent scalp flaking scale; SD, seborrheic dermatitis; SSA‐ZP, 2% supramolecular salicylic acid combined with 0.8% zinc pyrithione; TEWL, transepidermal water loss.

This self‐controlled clinical study spanned 2 weeks and was approved by Use Committees (IACUC) of the Third affiliated Hospital of Sun Yat‐Sen University ([2010]1‐7). Written informed consent was obtained from all patients. The research flowchart is presented in Figure 1.

### 2.2 Patient Selection

Five patients diagnosed with scalp SD by two experienced dermatologists separately. Inclusion criteria encompassed patients aged between 18 and 50 years, exhibiting scalp SD characterized by continuous mild to moderate itching, dandruff, and sebum secretion for at least 3 months. Exclusion criteria included a history of other scalp disorders such as psoriasis or contact dermatitis, recent oral or topical SD treatment within 1 month, known allergies to study medications or their components, or pregnancy or breastfeeding.

### 2.3 Treatment and Parameter Measurements

Patients were assessed 72 h post‐hair washing. Before treatment, patients acclimatized to a room maintained at 24°C–26°C for 30 min. The scalp was divided into six areas: left and right sides of the forehead, top of the head, and occiput. Dermoscopy (Guangzhou Chuanghong Medical Technology Corporation, China) photographs were collected for archiving. Parameters including sebum level (Sebumeter SM815, room temperature [24°C–26°C], and humidity [40%–45%]), transepidermal water loss (TEWL, Cutometer MPA 580, COURAGE+KHAZAKA, Germany), pH (Skin‐PH‐meter PH900, COURAGE+KHAZAKA, Germany), stratum corneum hydration (Corneometer CM825, COURAGE+KHAZAKA, Germany), and the fungal count were measured in each area using fluorescence microscopy (CX31, Olympus Corporation, Japan). Patients were evaluated using the adherent scalp flaking scale (ASFS), and the grading scale was as follows: Grade 0, no scales; Grade 1–2, a few small pieces of powdery gray thin scales; Grade 3–4, small to medium sized thin scales (mild); Grade 5–6, large, thin scales attached loosely to the scalp (moderate); Grade 7‐8, large adhesive thick scales (moderate–severe); and Grade 9‐10, thick yellowish‐white scales closely attached to the scalp (severe) [10].

SSA, ZP, and SSA‐ZP have received regulatory approval as a topical medication. Patients were asked to apply SSA‐ZP (Broda, Borenda Biochemical Technology, China) which consists of two active ingredients, 2% SSA and 0.8% ZP, and other auxiliary substrates including poloxamer 407, C12‐15 alkyl‐polyether‐4, polyethylene glycol‐8, glycerin, propylene glycol, lauryl polyether‐7, hydroxyethyl cellulose, sodium naphthalene sulfonate, and sodium lactate. SSA‐ZP was used gently to the entire scalp for 3–5 min until it was absorbed, once daily for 2 weeks. Indicators were assessed in six different scalp areas on days 1, 7, and 14 post‐treatments for itching, dandruff, sebum production, pH, TEWL, and the fungal count. Adverse reactions were recorded during the treatment. Patients were asked to get enough sleep and avoid alcohol and spicy foods, but they did not need to change their habits of hair washing or pillowcase cleaning, nor did they need any oral medications.

### 2.4 Statistical Analysis

Statistical analysis was conducted on data from six scalp areas, with results presented as mean ± standard deviation. Differences between means were verified using repeated measures analysis of variance. Analyses were performed using SPSS software (version 25.0; IBM Corporation, USA), with a *p* < 0.05 indicating statistical significance.

## 3 Results

Five patients with mild to moderate scalp SD were included in this study, including three males and two females aged 25–27 years. Following 2 weeks of SSA‐ZP treatment, all patients demonstrated a statistically significant improvement in their scalp condition (Table 1), with no apparent adverse reactions observed.

**TABLE 1:** The changes in average ASFS scores across different scalp areas from five patients (N1–N5) on days 1, 7, and 14 after treatment of SSA‐ZP conditioner (mean ± standard deviation).

Patient	Day 0	Day 7	Day 14	*p*
ASFS score				
N1	29.01 ± 2.65	20.67 ± 1.52	13.25 ± 0.96	< 0.01
N2	9.12 ± 2.66	5.33 ± 2.08	16.00 ± 1.15	< 0.01
N3	16.21 ± 2.13	9.33 ± 1.15	3.33 ± 0.57	< 0.01
N4	25.41 ± 2.08	17.03 ± 1.01	9.67 ± 0.57	< 0.01
N5	33.63 ± 1.02	20.67 ± 0.57	11.33 ± 0.58	< 0.01

Abbreviations: ASFS, the adherent scalp flaking scale; SSA‐ZP, 2% supramolecular salicylic acid combined with 0.8% zinc pyrithione.

### 3.1 SSA‐ZP Reduced the ASFS Scores and Effectively Treated SD Dandruff

**FIGURE 2:**
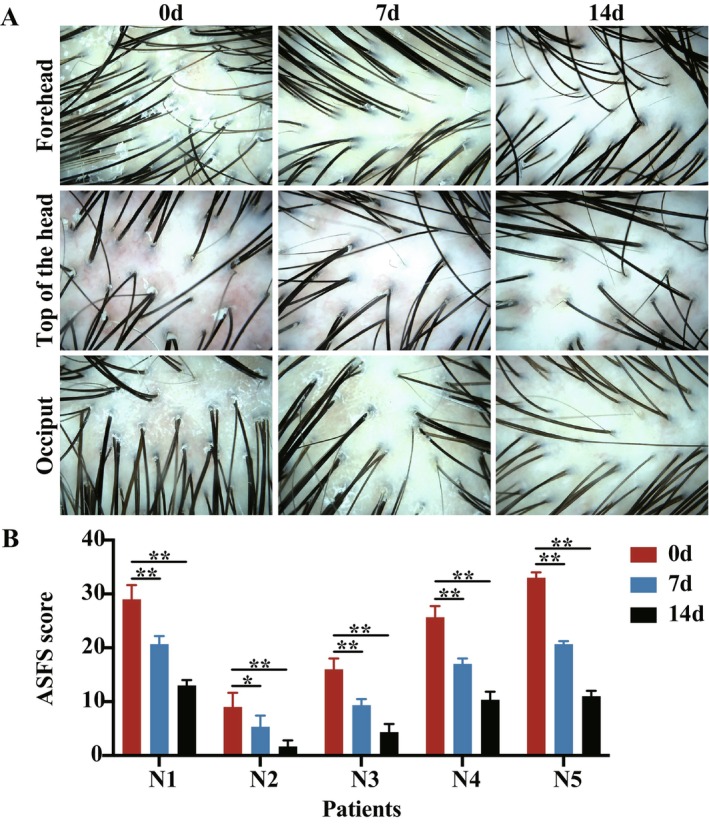
Effect of SSA‐ZP on ASFS scores and dandruff improvement of scalp SD. (A) Dermoscopy images of a 26‐year‐old male patient taken from the forehead, top of the head, and occiput on days 0, 7, and 14 posttreatment. (B) Average ASFS scores measured in six different scalp areas from five patients on days 0, 7, and 14 post‐treatment. ASFS, the adherent scalp flaking scale; SD, seborrheic dermatitis; SSA‐ZP, 2% supramolecular salicylic acid combined with 0.8% zinc pyrithione. *p < 0.05; **p < 0.01.

Dandruff was assessed using dermoscopy images and the ASFS scores. Based on dermoscopy images, a notable reduction in dandruff and erythema was observed in the forehead, top of the head, and occiput on days 0, 7, and 14 after treatment (Figure 2A). Compared with day 0, the average ASFS scores in different areas of scalp SD patients significantly decreased on days 7 and 14 after treatment (*p* < 0.01) (Figure 2B, Table 1), which were consistent with the results of dermoscopy. No apparent adverse reactions were observed in any patient.

### 3.2 SSA‐ZP Reduced Secreted Sebum Without Compromising Skin Barrier Function

**FIGURE 3:**
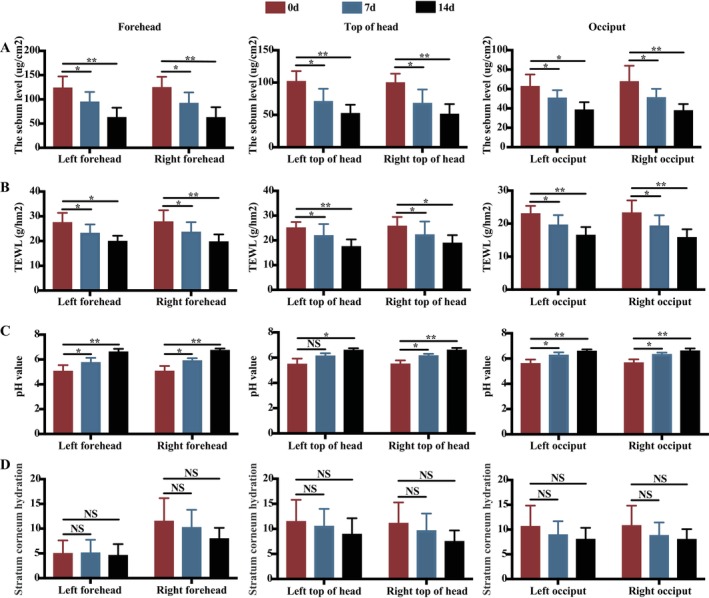
Measurement of scalp parameters. (A) Scalp sebum levels, (B) TEWL, (C) pH value, and (D) stratum corneum hydration measured on the left and right sides of the forehead, top of the head, and occiput on days 1, 7, and 14 posttreatment of SSA‐ZP. SSA‐ZP, 2% supramolecular salicylic acid combined with 0.8% zinc pyrithione; TEWL, transepidermal water loss. *p < 0.05; **p < 0.01.

We assessed the level of secreted sebum after SSA‐ZP treatment, which acts as one of the most important pathogenic mechanism of SD. Assessment of skin barrier function based on TEWL, pH, and stratum corneum hydration before and after treatment revealed a decrease in sebum levels (*p* < 0.01) (Figure 3A, Table 2) and TEWL (*p* < 0.01) (Figure 3B, Table 2), along with an increase in pH value (*p* < 0.01) (Figure 3C, Table 2), with statistical significance. However, after 14 days of treatment, no significant difference in stratum corneum hydration was observed (*p* > 0.05) (Figure 3D, Table 2). These results suggested that SSA‐ZP effectively reduces sebum secretion without compromising skin barrier function, and may even improve it.

**TABLE 2:** Measurement of scalp parameters including scalp sebum level, transepidermal water loss (TEWL), pH value, and stratum corneum hydration for the left and right sides of the forehead, top of the head, and occiput on days 1, 7, and 14 after treatment of SSA‐ZP conditioner (mean ± standard deviation).

	Day 0	Day 7	Day 14	*p*
Sebum level (μg/cm^2^)				
Left forehead	123.05 ± 0.01	94.25 ± 18.77	62.20 ± 18.41	0.0092
Right forehead	103.29 ± 20.14	77.54 ± 20.17	54.08 ± 19.39	0.0068
Left top of head	84.63 ± 14.44	60 ± 17.73	45.67 ± 12.21	0.0029
Right top of head	82.96 ± 12.59	57.58 ± 19.35	44.75 ± 14.14	0.0042
Left occiput	52.08 ± 11.18	43.25 ± 7.44	34.23 ± 7.24	0.0068
Right occiput	56.21 ± 14.79	43.60 ± 8.24	33.63 ± 6.16	0.0068
TEWL (g/hm^2^)				
Left forehead	22.86 ± 3.54	20.43 ± 3.23	18.88 ± 2.05	0.0298
Right forehead	23.10 ± 4.23	20.80 ± 3.63	18.72 ± 2.71	0.0601
Left top of head	20.81 ± 2.17	19.39 ± 4.23	16.87 ± 2.62	0.0214
Right top of head	21.41 ± 3.34	19.67 ± 4.85	18.03 ± 2.92	0.1182
Left occiput	19.15 ± 2.14	17.46 ± 2.71	16.05 ± 2.24	0.013
Right occiput	19.36 ± 3.40	17.23 ± 2.91	15.46 ± 2.27	0.0128
pH value				
Left forehead	5.05 ± 0.45	5.74 ± 0.35	6.60 ± 0.23	0.0001
Right forehead	5.05 ± 0.38	5.89 ± 0.19	6.72 ± 0.14	< 0.0001
Left top of head	5.48 ± 0.40	6.12 ± 0.20	6.58 ± 0.14	< 0.0001
Right top of head	5.49 ± 0.26	6.15 ± 0.14	6.59 ± 0.16	< 0.0001
Left occiput	5.60 ± 0.29	6.27 ± 0.20	6.58 ± 0.13	0.0004
Right occiput	5.66 ± 0.25	6.32 ± 0.14	6.60 ± 0.18	0.0001
Stratum corneum hydration				
Left forehead	4.13 ± 2.39	5.40 ± 2.38	6.14 ± 2.05	0.1646
Right forehead	9.58 ± 4.17	9.67 ± 3.23	8.95 ± 1.98	0.224
Left top of head	9.55 ± 3.88	9.93 ± 3.11	9.76 ± 2.87	0.4969
Right top of head	9.24 ± 3.75	9.19 ± 3.07	8.56 ± 1.99	0.177
Left occiput	8.85 ± 3.77	8.60 ± 2.46	9.00 ± 2.09	0.177
Right occiput	8.98 ± 3.63	8.48 ± 2.37	9.00 ± 1.85	0.2157

Abbreviations: ASFS, the adherent scalp flaking scale; SSA‐ZP, 2% supramolecular salicylic acid combined with 0.8% zinc pyrithione; TEWL, transepidermal water loss.

### 3.3 SSA‐ZP Demonstrated Strong Antifungal Activity

**FIGURE 4:**
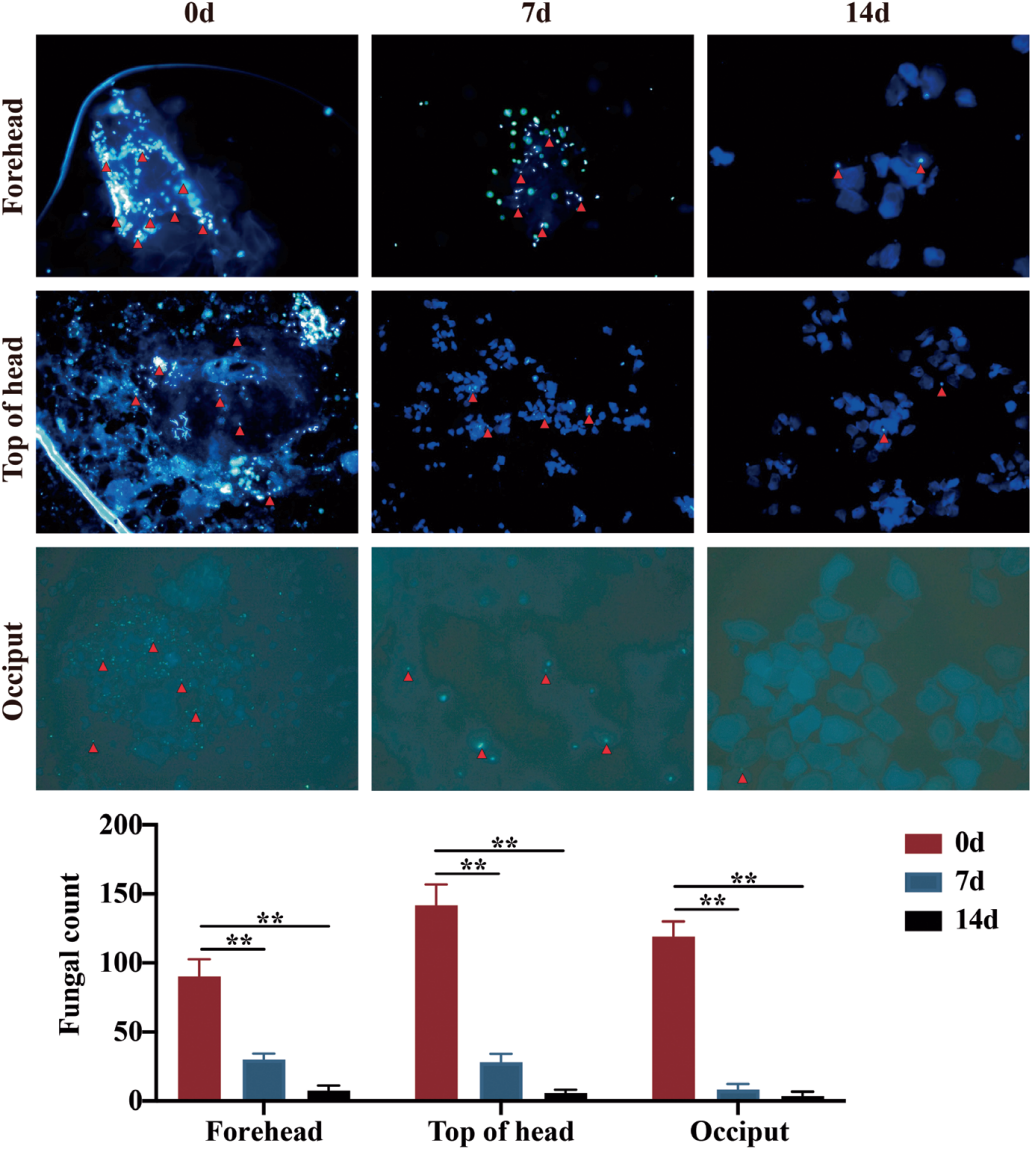
Reduction in fungal count with SSA‐ZP treatment across various scalp areas on days 1, 7, and 14 posttreatment. The photos (D0, D7, and D14) were taken from the same patient (×400 magnification). The red arrows point to the fungal spores (small round spots of bright blue) distributed individually or in clusters. SSA‐ZP: 2% supramolecular salicylic acid combined with 0.8% zinc pyrithione. **p < 0.01.

Fungal infection, particularly Malassezia spp., is a key factor in scalp SD development. ZP, a traditional antifungal agent, has been shown to inhibit most scalp skin Malassezia species, including *Malassezia globosa* and *Malassezia restricta*. However, its efficacy depends on the targeted delivery system of ZP to infected skin [9]. To assess the efficacy of SSA combined with ZP, we used fluorescence microscopy to quantify fungi spores before and after treatment. The results revealed a significant decrease in the number of fungal spores across all six scalp areas after 14 days of treatment (*p* < 0.01) (Figure 4).

## 4 Discussion

Scalp seborrheic dermatitis (SD) presents a considerable clinical challenge due to its common occurrence and profound impact on patients' quality of life [11]. Dandruff, a typical symptom of SD, is widely believed to result primarily from microbial dysbiosis, with Malassezia yeast species, particularly *M. restricta* and *M. globosa*, playing a pivotal role in its pathogenesis [12].

Since its synthesis in the 1950s, zinc pyrithione (ZP), a well‐established antifungal agent, has been a cornerstone in the treatment of scalp SD with a long‐term safety [13, 14]. ZP's mechanism of action entails its penetration into yeast cells via pyrithione ionophore, including cellular stress, disrupting energy metabolism, and reducing lipase expression, ultimately exerting antifungal effects. While reports of fungal resistance to ZP are scarce, its low aqueous solubility (5–15 ppm) restricts skin permeation, typically resulting in < 0.05% of the initially applied dose reaching the target site [11]. Currently, ZP is primarily used in shampoo or lotion formulations, which presents a significant drawback due to their short contact time and low concentration on the scalp, particularly in regions beyond the skin surface and superficial follicle [15], where adequate ZP for antifungal effects may not be achieved [11]. These factors lead to the limited bioavailability of ZP. Furthermore, the multifactorial nature of scalp SD, involving inflammation, sebum production, and barrier impairment [16], underscores the necessity for a comprehensive treatment approach beyond ZP monotherapy.

Salicylic acid (SA), also known as 2‐hydroxybenzoic acid, possesses anti‐inflammatory properties and mild antimicrobial effects [17]. Recent studies have shown that shampoos or lotions containing 1.3%–3% SA effectively alleviate symptoms of scalp SD such as dandruff, erythema, and itching [18]. How to reduce sebum secretion is an important focus in scalp SD treatment. However, the literature presents conflicting findings regarding SA's ability to inhibit sebum secretion due to its lipophilic nature. For instance, while Lee [19] found no significant difference in sebum levels following SA application for facial acne, Lu et al. [20] demonstrated SA's inhibitory effect on sebum formation by downregulating the expression of SREBP pathway. Supramolecular salicylic acid (SSA) emerges as a promising advancement, which preserves SA's pharmacological effects while offering slow‐release action and reduced side effects [21]. Thirty percent SSA has been shown to have a good clinical safety after 15 months treatment of facial SD [22]. Nonetheless, prior investigations have primarily focused on the role of 30% SSA in the treatment of acne [23, 24] and facial SD [22], leaving a gap in understanding the potential application of 2% SSA in scalp SD treatment. Furthermore, a study showcased the dual benefits of 30% SSA in reducing facial sebum secretion and improving the skin barrier function [5].

Motivated by these findings, the innovation of our study is to fill the gap of clinical research of the combination of 2% SSA and 0.8% ZP in scalp SD, and to evaluate the efficacy and safety of SSA‐ZP in the treatment of scalp SD with comprehensive and objective indicators, particularly assessing its impact on sebum levels and scalp barrier function. Our results showed that SSA‐ZP conditioner effectively reduced sebum production, ASFS scores, dandruff severity, and fungal count at both 7 and 14 days posttreatment. Moreover, although no difference in the hydration was observed, scalp barrier function was enhanced, as evidenced by the acidic pH value and improved TEWL. Various investigations revealed that patients with SD may profit from washing with acidic syndets because of the reduction of fungal infection and favorable tolerability profile [25]. The acidic skin surface play an important role in the keratinization and barrier regeneration [26]. SSA‐ZP is a kind of topical acidic buffer substances applied to the scalp. The results of our study showed that after the treatment of SSA‐ZP, although the pH value of scalp skin was increased, it was still in the acidic range, suggesting that SSA‐ZP had a protective effect on the skin barrier, which is consistent with the results of Lingzhao Zhang et al. [5]. These findings suggest that SSA enhances ZP's antifungal action by enhancing its anti‐inflammatory properties and improving scalp barrier function. The combination of SSA and ZP targets multiple pathogenic factors of scalp SD, leading to satisfactory clinical efficacy and safety. Furthermore, our preliminary study found significant improvements in clinical manifestations and laboratory indicators after only 2 weeks treatment with SSA‐ZP, which may provide a reference time for observation of the efficacy of SSA‐ZP in the treatment of scalp SD. As our study only included five patients, which is a limitation of this paper, additional studies involving larger sample sizes and extended follow‐up periods are warranted to confirm the sustained effectiveness and safety of SSA‐ZP combination therapy.

## 5 Conclusion

The combination of SSA with ZP in treating scalp seborrheic dermatitis presents a compelling therapeutic approach. By leveraging the self‐assembly properties of SSA, this formulation enhances the solubility and bioavailability of both active ingredients.

This synergistic combination not only targets multiple aspects of SD—such as inflammation, flaking, and fungal growth—but also offers a controlled release mechanism. This can minimize potential side effects associated with conventional treatments and improve patient compliance by reducing the frequency of application needed to manage symptoms effectively. Overall, the integration of supramolecular technology with these active ingredients holds promise for a more effective and patient‐friendly approach to managing scalp SD, and offers new treatment options to improve other skin inflammation.

## Author Contributions

Q.W. and Y.L. collected the data and drafted the manuscript, contributing equally to this work. C.Y. analyzed the data. J.S. and J.L. assisted in the revision of the manuscript. Y.D. and L.L. assisted in the proofreading of the manuscript and the responses to the reviewers' comments. X.H. and Y.Z. designed the study and revised the manuscript. All authors have read and approved the final manuscript.

## Ethics Statement

The experiments involving human participants were reviewed and approved by Use Committees (IACUC) of The Third Affiliated Hospital of Sun Yat‐Sen University ([2010]1‐7).

## Consent

The patients provided their written informed consents to participate in this study.

## Conflicts of Interest

The authors declare no conflicts of interest, neither financial or nonfinancial, with the company manufacturing or marketing SSA‐ZP products (Borenda Biochemical Technology).

## Data Availability

The data that support the findings of this study are available from the corresponding author upon reasonable request.
